# The “Supporting Adolescents with Self Harm” (SASH) Intervention Supporting Young People (And Carers) Presenting to the Emergency Department with Self-Harm: Therapeutic Assessment, Safety Planning, and Solution-Focused Brief Therapy

**DOI:** 10.3390/healthcare14020168

**Published:** 2026-01-08

**Authors:** Rose McCabe, Sally O’Keeffe, Maria Long

**Affiliations:** 1 School of Health and Medical Sciences, City St. George’s, University of London, London EC1R 1UW, UK; 2 Population Health Sciences Institute, Newcastle University, Newcastle upon Tyne NE1 7RU, UK

**Keywords:** self-injurious behaviour, suicide prevention, adolescent, emergency service, crisis intervention, carers

## Abstract

**Background**: Self-harm is a growing public health concern and the strongest predictor of suicide in young people (YP). The “Supporting Adolescents with Self-Harm” (SASH) intervention was developed with YP with lived experience and expert clinicians. It involves rapid follow-up after ED attendance and up to six intervention sessions. The intervention has three components: Therapeutic Assessment (TA) of self-harm; an enhanced safety plan (SP); and Solution-Focused Brief Therapy (SFBT). Depending on the YP’s preference, carers can join sessions. Carers can also receive two individual sessions. The clinical and cost-effectiveness of SASH is being evaluated in a randomised controlled trial across nine emergency departments in three NHS Trusts in London, England. A total of 154 YP were recruited between May 2023 and March 2025 and randomised on a 1:1 ratio to SASH alongside Treatment As Usual (TAU) or TAU. A logic model describes the SASH inputs, activities, mechanisms, outcomes and longer-term impacts. The aim of this paper is to (1) illustrate how TA, SP, and SFBT were implemented in practice by presenting intervention materials and session recordings for four YP cases and one carer case and (2) explore how the case study materials/recordings reflect the intervention mechanisms in the SASH logic model. **Methods**: Each case focused on a different component of the intervention. Intervention materials (TA self-harm diagram and completed SP) and recorded SFBT sessions with four YP and one carer were analysed using a descriptive case study approach. The TA diagram and SP were extracted from medical records. Audio/video recordings of intervention sessions were identified. Recordings of intervention sessions and qualitative interviews were transcribed. Quotes from qualitative interviews with the same participants were included where relevant. **Results**: Across the four YP cases, some core themes emerged. The role of friendships for young people, particularly at school, was important in both negative and positive ways. Experiencing difficulties with friends at school led to feelings of sadness and stress, which could become overwhelming, leading to thoughts of self-harm (“I just need to hurt myself”), triggering self-harm behaviour. YP described mood changes and signs that they were becoming stressed, which improved their self-awareness and understanding of the link between their feelings and self-harm behaviour. They reflected on what kept them feeling calm and overcoming their fear of burdening others by sharing how they were feeling, as this helped them not to self-harm. They also described difficult feelings stemming from a need to please everyone or needing validation from others. Overcoming these feelings led to less social anxiety and more confidence. This made it easier to go to school and to be more social with friends/student peers, which in turn improved their mood. **Conclusions**: These case studies demonstrate how YP improved their self-awareness and understanding of the link between feelings and self-harm behaviour and identified personal strategies for managing difficult feelings and situations. The carer case study demonstrates how sessions with carers can facilitate carers better supporting their YP’s mental health. Supporting YP and carers in this way has the potential to reduce the risk of future self-harm.

## 1. Introduction

Self-harm is a growing public health concern. Self-harm is defined as any “intentional self-poisoning or injury irrespective of the apparent purpose of the act” [1]. The 2023/24 UK government figures show that in young adults (ages 16–24 years old), 31.5% reported lifetime suicide thoughts, 24.6% reported having self-harmed and 10.3% reported a lifetime suicide attempt [2]. Self-harm is the strongest risk factor for suicide in young people (YP), which is one of the leading causes of death in YP [3]. Over 50% of those under 20 who die by suicide have a history of self-harm [4]. YP who visit the emergency department (ED) with self-harm have up to a 50-fold risk of future suicide. This makes the ED a crucial opportunity for intervening as early as possible with YP presenting with self-harm.

In England, the National Institute for Health and Care Excellence (NICE) recommends a psychosocial assessment by specialist mental health practitioners in the ED for YP who present with self-harm and a 7-day follow-up [1]. Practitioners from Child and Adolescent Mental Health Services (CAMHS) crisis teams conduct psychosocial assessments to assess mental health, risks, care needs, and agree on a management plan. Follow-up within 7 days seeks to provide appropriate aftercare, including reviewing the risk and onward referrals. However, evidence from a meta-synthesis found that YP often report that ED treatment for self-harm exacerbates their distress and that EDs are unable to meet their needs [5]. Difficult experiences of ED deter future help-seeking and contribute to vicious cycles of repeat ED attendances and escalating suicidality [6]. A recent review found that the international picture is consistent with that in the UK; globally, many YP receive neither assessment in the ED nor follow-up, and only around half are referred on for further input from community mental health services [7].

While there are few psychological interventions for YP presenting to the ED with self-harm, different activities have been investigated. A novel approach to the assessment of self-harm in YP is Therapeutic Assessment (TA). It involves describing the YP’s personal cycle of self-harm to help the YP understand their emotions and self-harm behaviour, along with possibilities for breaking the cycle [8]. TA is a promising approach that is therapeutic in seeking to understand the complex reasons and psychological “core pain” that drives self-harm in young people. A previous non-randomised study found that YP who received TA were more likely to engage in follow-up care after attending the ED and less likely to harm themselves [9].

Safety plans (SPs) have been investigated and found to be effective for suicide prevention in adults. However, research on safety plans with YP is limited [10]. A non-randomised study evaluating an intervention that included safety planning in a mixed sample of adolescents partially recruited from emergency departments saw reductions in suicidal ideation and behaviour at 6-month follow-up [11]. Support for YP after discharge is lacking due to underfunding and increasing rates of CAMHS referrals [12]. As a result, many YP who have presented to the ED with self-harm may require further support and are referred to CAMHS but end up on lengthy waiting lists for treatment: YP are typically waiting over one year for CAMHS treatment in the UK [13]. The gap between demand and availability of services is an international problem, as data from North America reveal a similar picture of YP facing lengthy waits to access treatment [14]. Long wait times prolong distress and exacerbate YP’s mental health difficulties [15]. A recent report found that 26% of YP attempted suicide while waiting for CAMHS treatment [16]. Deteriorating mental health also has wider impacts on school absence and poorer social and educational development.

Solution-focused brief therapy (SFBT) is a strengths-based intervention where YP are considered experts in their own lives. In other settings, SFBT is as effective as other evidence-based treatments, but progress happens in fewer sessions: This requires fewer resources, making it potentially more cost-effective (average 4–5 sessions, compared to 8 for other talking therapies) [17,18]. As it is a brief systemic approach, it has the potential to support YP and families in a mental health crisis.

To address the current gap in rapid interventions for YP in crisis, we developed the Supporting Adolescents with Self-Harm (SASH) intervention with the input of YP/carers with lived experience and mental health clinicians. The SASH intervention aims to provide rapid follow-up within 1–2 weeks after attending the ED. It includes up to six sessions over approximately 2 months with YP and parents/carers. The intervention comprises the following: (1) TA of self-harm, addressing the YP’s core pain, their cycle of self-harm, and identifying how to break this cycle; (2) an enhanced SP based on the Stanley and Brown model; and (3) SFBT where the YP/carer identifies their future hopes and resources to help them move forward in their lives. Depending on the YP’s preference, parents/carers are invited to join sessions with the YP. Carers can also receive two individual sessions focused on psychoeducation around self-harm and their best hopes for themselves and their child. Evidence shows that involving families and the system supporting the YP improves outcomes for YP [18].

The clinical and cost-effectiveness of the SASH intervention in addition to TAU is currently being evaluated in a randomised controlled trial in England. It is being compared to Treatment As Usual (TAU), and the primary outcome is repeat self-harm after 6 months. Full details are described in the trial protocol [19] (https://sashstudy.co.uk/, accessed on 19 December 2025). Secondary outcomes include symptoms of depression and anxiety, ED reattendance, death by suicide, school attendance, and psychological wellbeing (see [19] for all outcomes), collected at baseline and 6-month follow-up. A total of 154 YP were recruited into the trial and are currently being followed up, with follow-up data collection having recently been completed. SASH draws on existing components of self-harm interventions that have been tested in previous studies, including Therapeutic Assessment (TA), safety planning, and SFBT. The aim of this paper is to (1) illustrate how TA, safety planning, and SFBT were implemented in practice by presenting intervention materials/recordings for four YP cases and one carer case and (2) explore how the case study materials/recordings reflect the intervention mechanisms in the SASH logic model. A future publication will report on the clinical and cost-effectiveness of the SASH intervention.

## 2. Materials and Methods

### 2.1. Setting

We present intervention materials and recordings from the SASH trial. SASH is a multicentre parallel group individually randomised controlled trial (RCT). YP and carers were recruited across nine ED departments in west, north, central, and east London. The trial has been described in full elsewhere [19]. CAMHS practitioners working with YP in crisis were trained in the SASH approach across 1.5–2 days. A manual, training videos, and handouts were provided to practitioners to support the delivery of the intervention and adherence to the approach. Practitioners were invited to weekly group supervision with senior members of the research team to discuss cases and discuss the delivery of the intervention in line with NHS Trust clinical processes and policies.

### 2.2. The Intervention

The intervention comprises up to six sessions with YP and their carers. The first session is delivered within approximately seven days of ED attendance. Subsequent sessions are delivered at approximately two-week intervals. Sessions are primarily delivered face-to-face, though remote delivery is possible where permitted by clinical services. Intervention delivery is designed to be flexible to maximise engagement. In response to changes in risk or worsening of presentation, clinicians can refer YP on for more support or for specialist assessment. The first session includes TA and safety planning, while follow-up sessions are SFBT sessions. To ensure consistency in intervention delivery across sites, the research team met regularly with practitioners (aiming for every two weeks), encouraged attendance at weekly supervision, and provided top-up training in specific areas (e.g., working with parents/carers). To assess intervention fidelity, two researchers independently applied a bespoke fidelity scale to a sample of practitioner intervention session notes. Bespoke logs were developed to monitor adherence to the intervention and record practitioner supervision attendance. We worked with senior leadership teams in services to support practitioner attendance at supervision alongside their clinical commitments.

### 2.3. Therapeutic Assessment

TA was developed by Ougrin et al. specifically for YP presenting with self-harm in crisis [8]. It is a novel approach to working with YP (and families), derived from solution-focused and systemic traditions. It involves joint production of a diagram of the self-harm cycle, with three elements: identification of core pain, maladaptive behaviours, and means to break the cycle. YP are asked to identify a previous episode of self-harm. Diagrams are constructed using the YP’s language and aim to identify and understand the YP’s difficulties, instil hope, and provide a foundation for therapeutic change. See Figure S1 in the Supplementary Materials for a schematic of the TA diagram.

### 2.4. Enhanced Safety Planning

The SP is an adapted version of the Stanley and Brown model of safety planning [20] and involves co-construction of an individualised safety plan in the YP’s own words. This consists of two sections: the first section describes the YP when they are well and what they like to do, what helps them to stay well, and what might get in the way of this. The second section relates to warning signs of self-harm and means of managing thoughts of self-harm, including individual actions (distractions and changing environment), and support networks that can be drawn on (contacting trusted others and professionals). The SP can be in paper or electronic form and can be completed either by the YP, practitioner, or jointly. For enhanced safety plan template, see Figure S2.

### 2.5. Solution-Focused Brief Therapy

The follow-up sessions (up to five) involve SFBT. SFBT is a strengths-based intervention involving a paradigm shift from problem reduction to promoting strengths and new ways of thinking and behaving. SFBT is as effective as other evidence-based treatments but is achieved in fewer sessions (average 4–5 sessions). A systematic review and meta-analysis found that SFBT is effective for YP and adult depression, low self-esteem, family dynamics, relationship problems, and parental stress in community settings [21]. SFBT for carers has been found to improve YP mental health [22]. SFBT’s focus on the therapeutic relationship empowers YP to experience a more hopeful future through collaboratively formulating solutions and how to move forward [23,24,25,26]. For SFBT templates, see Figures S3 and S4.

### 2.6. Parent/Carer Involvement

According to the YP’s preference and consent, parents/carers may join sessions with the YP. Parent/carer involvement can support the YP emotionally, improve their understanding of the YP’s experience, and increase awareness of strategies that can be used by the YP. Parents/carers may be invited to participate in a joint SFBT session with the YP, where a shared understanding of what the YP and parent/carer may be generated. In addition to joining the sessions with their YP, parents/carers are offered 1–2 of their own individual sessions (these may involve more than one parent/carer) where clinicians take a solution-focused approach and offer psychoeducation for self-harm. We will explore the effects of parent/carer involvement systematically in statistical analyses in the randomised controlled trial.

### 2.7. Data Collection

#### 2.7.1. Participants

Participants were identified from the group of YP in the SASH intervention arm (*n* = 78), who had either intervention session recordings available or intervention-related materials uploaded to their medical records. Parents/carers of YP in the intervention arm were selected if they had an individual or joint intervention session recording available.

#### 2.7.2. Procedure

Participants provided informed consent for audio/video recording for intervention sessions and qualitative interviews and specified consent for the use of this data as part of consent for participating in the trial. Audio/video files were transcribed using a two-step procedure; firstly, a secure auto-transcription tool was used to capture the live interaction and was downloaded, after which auto-transcripts were manually checked and corrected for accuracy. Before recording, practitioners reaffirmed the participants’ consent to be recorded. Recordings were made on secure, encrypted devices and were securely transferred to approved electronic storage. All identifying details were removed from transcripts, and participants were assigned pseudonyms.

#### 2.7.3. Sampling

1. Intervention sessions and documents

Of the pool of young people assigned to the intervention arm (*n* = 78), we reviewed all available intervention session transcripts (*n* = 16) and intervention-related materials (TA diagrams *n* = 7, SPs *n* = 8, SFBT handouts *n* = 16). The intervention manual and fidelity scale were used to guide the selection of cases with acceptable fidelity to the intervention manual. Selections were made by reviewing transcripts of intervention sessions and intervention materials to identify exemplars of each of the three intervention components. We also aimed to capture variation across the type of session (YP only and parent/carer session only). All data were anonymised prior to analysis. Assessments of fidelity to the intervention will be published alongside the main trial results.

2. Qualitative interviews

Purposive sampling was used in the SASH trial to invite YP from the intervention arm and practitioners who delivered the intervention to take part in a qualitative interview as part of the process evaluation. Full transcripts were reviewed, and excerpts were identified that corresponded to that person’s TA, SP, and SFBT sessions. Practitioner qualitative transcripts were reviewed for reflections on the implementation of the intervention relating to the activities and mechanisms of change.

### 2.8. Data Analysis

A descriptive case study approach [27] was taken when analysing the intervention materials and recordings. Activities and mechanisms of change from the SASH logic model were used as a framework to guide the presentation of each component of the intervention.

**Figure 1:**
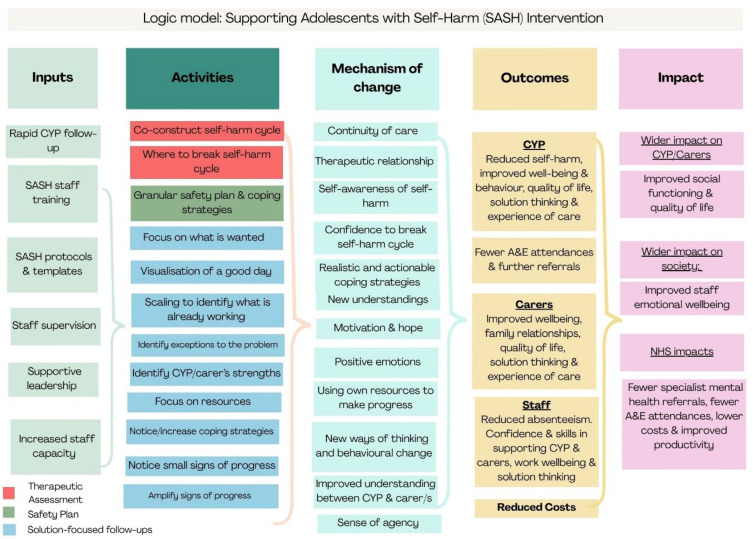
SASH intervention logic model.

The logic model in Figure 1 was developed based on existing evidence of how the different components of the SASH intervention are intended to influence outcomes, informed by literature review, expert input, and refined iteratively during intervention delivery. Inputs, activities, mechanisms of action, outcomes, and impact outline the theory of change underpinning the intervention. Inputs relate to resources and assets which support the intervention, for example, practitioner training and supervision; activities relate to actions or processes which are both specific to the different components of the intervention and generic across the approach; mechanisms of change relate to how and why the intervention achieves its intended outcomes; outcomes relate to the intended consequences of the intervention and impact relates to the longer term effects of the SASH intervention on key stakeholders. In the trial, we administered a measure of the quality of the therapeutic relationship post-intervention (or TAU), as we hypothesise that this outcome may mediate the effect of the intervention. Other mechanisms in the logic model will be explored in qualitative and quantitative analyses.

### 2.9. Ethics

The SASH study was approved by the London-Riverside Research Ethics Committee (Ref: 22/LO/0400). Fully informed written consent was sought from YP, parents/carers, and practitioners. For YP under the age of 16 years, written assent was sought in addition to fully informed written parental consent. YP, carer, and practitioner consent forms included optional items for audio/video recording of sessions: When this optional consent was obtained from all parties, sessions were recorded where possible by the practitioner. Pseudonyms have been used and identifying details changed to preserve anonymity in the presentation of the case studies.

## 3. Results

Five case studies are presented, as shown in Table 1. The case studies include four YP who received the SASH intervention, each focusing on a different component of the intervention (TA, SP, and SFBT) and one parent/carer (mother) of a YP who received the SASH intervention.

### 3.1. Case Study 1: Therapeutic Assessment of Self-Harm with Adah

In most cases, the TA was conducted in person in the first session, within one week of ED attendance. As the SASH intervention was provided in addition to TAU, this session also typically included a reassessment of risk, mental state, and a check-in around referrals and contact with other agencies, e.g., social care, which is what would be provided in the TAU routine 7-day follow-up. The practitioner report suggests that TA took longer on average with early teens and with YP who are neurodivergent, taking longer to identify feelings and link behaviours together in a chain. Practitioners could use the “Feelings Wheel” [28] from the intervention manual to help YP identify emotions, consider relevant adaptations such as using emoji scales instead of numbers, and were advised to spend additional time building rapport with YP if needed.

**Figure 2:**
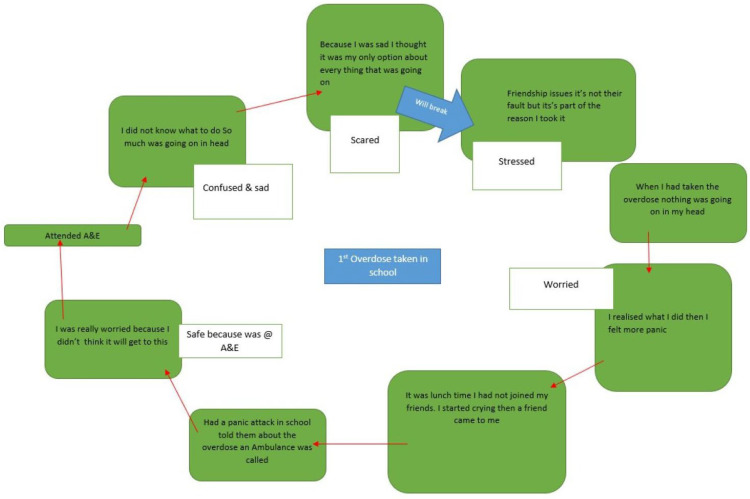
Self-harm cycle for Adah.

Case study 1 focuses on the TA of Adah, who presented to the ED with a suicide attempt. She received five sessions in total across 3 months. During the first session, the YP and practitioner *co-constructed the self-harm cycle* (see Figure 2). The YP began by describing the events leading up to her suicide attempt and explained that she had been feeling stressed and sad (core pain) while at school due to issues in her friendship group (trigger) and felt the only way to deal with these overwhelming feelings was to take an overdose. When she took the overdose, she described experiencing a clear mind (short-term relief). She then started to reflect on what she had done, felt panic and worry, started crying, and had a panic attack. This led to disclosure about the overdose to school staff, who called an ambulance. When she reached the hospital, she felt safe because she was in the ED but described feeling worried about herself and what she had done, which led to feelings of confusion and sadness and disbelief at her actions (long-term negative consequences). She identified that she would choose to *break the self-harm cycle* by noticing when she is feeling stressed or has mixed emotions, for example, after friendship conflict, and identified a range of things as part of safety planning that she can do instead of self-harming, building a sense of *confidence in alternative means of coping*.

### 3.2. Case Study 2: Enhanced Safety Planning with Eliana

In most cases, the practitioner and YP conducted safety planning in the first session after completing the TA cycle. In some situations where more time was needed, the YP took the safety plan home to complete, while others completed it at the beginning of the second session. In routine care (or Treatment As Usual in the trial), a SP is discussed in the psychosocial assessment in the ED. SASH practitioners were advised to incorporate any previous elements of the ED safety plan into the SASH SP where relevant. Parents/carers were often brought into the session to discuss the safety plan with the practitioner and YP to support its use.

Case study 2 focuses on Eliana, who attended five sessions (one TA/SP session and four SFBT follow-up sessions). Eliana’s issues were centred around school—they were not regularly attending school and found it difficult when they did. They were being seen by a broad range of professionals, including a family therapist.

The first section of the SP involves getting to know the YP, exploring what they are like when they are well and what they like to do, what helps them to stay well, and what might get in the way of this. This begins to build a picture of the YP’s life, where they feel safe, who they trust, and what they feel their barriers are to feeling well (difficulties in their life). Getting to know the YP fosters a *therapeutic relationship* and helps to personalise the intervention. This YP commented on its comprehensiveness: “I liked how it’s more detailed and I was able to add more to the plan rather than the one in the A&E one where it was just like more basic….”. SPs in the ED tend to focus on means restriction and generic signposting to crisis lines/support.

In the first section of the SP, the YP identified positive attributes of themselves, to begin to engender *positive emotions* and *identify their own strengths*. They described themselves as loud, confident, fun, easy to talk to, caring, reliable, funny, loyal, honest, and beautiful when they feel okay. When feeling okay, they described their preferred activities as drawing/art, listening to music, watching TikTok/shows, spending time with cousins, and taking a nap. To feel okay, the YP described that she needed sleep, to watch comfort shows, TikTok, and to talk to cousins on the phone. Reflecting on this helps the YP to begin to recognise that they can *use some of their own resources to make progress*. Barriers to feeling okay were identified as arguments, [feeling] physically unwell, bad relationships with friends, not being able to regulate emotions, and being around too many people.

Practitioners then support the YP to create a *granular safety plan with multiple coping strategies*. Eliana began by identifying warning signs of escalating distress, *building self-awareness of self-harm*, and the onset of thoughts of self-harm. Practitioners are encouraged to use a traffic light system to help the YP identify early warning signs before self-harm becomes too challenging to avoid. Eliana identified physical sensations (feeling extremely hot, head twitching slightly, mini panic attacks, fidgeting, picking fingernails, squeezing the palms of hands, negative thoughts (worthlessness, failure, and “don’t deserve to be here”), and emotional changes (sudden mood changes and angry). Elian was usually in her bedroom or in public when this started and described a range of ways to distract herself: by watching a comforting show on Netflix, listening to music, drawing, and spending time with cousins. She felt she was likely to do this only sometimes, as she would be thinking, e.g., “I just need to hurt myself” and that her distress may burden others who are close to her. Eliana identified small steps to overcome these barriers, building *motivation and hope,* and ensuring the *strategies are realistic and actionable*. Small steps included managing low self-esteem with positive affirmations, building her confidence more by speaking to her parents, and talking to others. She noted new ways of thinking about herself and the importance of building herself up: “I can’t please everyone, therefore the best person I can please is myself”, suggesting a developing *sense of agency*.

YP then consider *ways to break the cycle of self-harm* by distracting themselves from escalating distress, specific to the setting in which the warning signs typically emerge. This YP identified walking in the local area, going to a place they like, such as the cinema or their cousin’s house, or going to their bedroom. They described it as being very likely that they would use these techniques, but that feeling too distressed/overwhelmed, not wanting to ruin the fun, or pressure from the group might stop them. Small steps to overcome these barriers included improving self-esteem and confidence, limiting social anxiety, reading motivational quotes, and using countdown techniques.

In the final two sections of the SP, YP are prompted to *use their own resources to make progress* by identifying others whom they can contact as an alternative way of *breaking the cycle of self-harm* and increasing their *sense of agency*. This YP identified parents and family (cousins) as people to contact when feeling overwhelmed but noted that it was not very likely that they would contact them because they would feel like a burden, unheard, and judged. Small steps to overcome these barriers would be if close others were willing to listen and be understanding.

In the final stage, YP identify professionals whom they would contact when in distress, in order to build awareness of available *realistic and actionable coping strategies*. The YP described that they would be very likely to access a mixture of school (school counsellor and safeguarding network), healthcare (GP, A&E, and crisis line), and charity-based (Kooth, Young Minds, and Textline) professional support, but barriers would include the fear of being misunderstood or unheard. The YP reported that identifying what they would discuss and imagining that the professional would be a listening ear would be ways to overcome these barriers.

### 3.3. Case Study 3: First Solution-Focused Brief Therapy Session with Charlotte

The first SFBT session follows the therapeutic assessment/safety plan session. Given the recent presentation to the ED with self-harm, this tended to also include a check-in on any self-harm/suicidal ideation or safeguarding issues that may have needed attention since the previous session.

The following session involves Charlotte, a girl, aged 14, who presented to the ED with suicidal thoughts (with recent self-harm). She was not attending school regularly.

- Best Hopes

**Figure 3:**
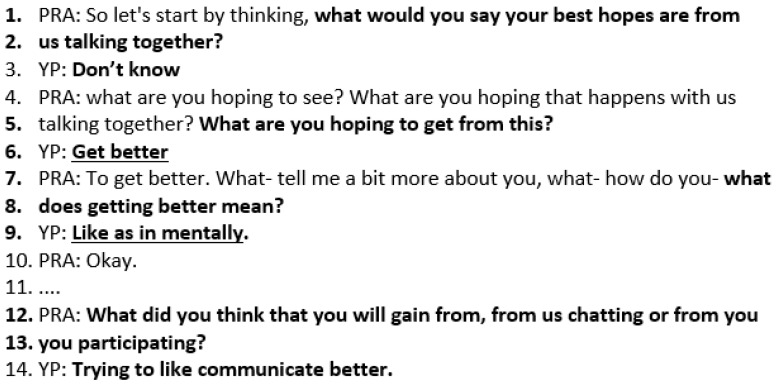
Transcript excerpt from Charlotte’s SFBT session: exploring best hopes.

The practitioner asks the opening question about *what is wanted by the YP:* “what are your best hopes from us talking?”. Charlotte answers, “I don’t know”. Often, YP respond in this way. This is permission to ask another question. The practitioner asks again in lines 4–5, “what are you hoping to see?”, and Charlotte responds, “Get better”. The practitioner asks for clarification (lines 7–8), “what does getting better mean?”, and Charlotte responds, “as in mentally”. The practitioner asks for some further information (lines 12–13), and Charlotte responds, “trying to communicate better”. This shows how the focus is on what Charlotte hopes to achieve (underlined text) rather than on the practitioner’s agenda. This increases engagement in the session as it defines the agenda for the whole session. It moves the talk away from the problem to what is wanted. In this session, Charlotte hopes to feel mentally better and to communicate better (Figure 3).

**Figure 4:**
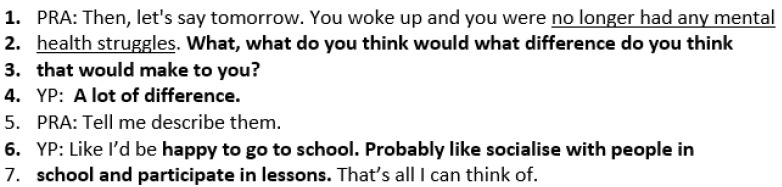
Transcript excerpt from Charlotte’s SFBT session: the difference achieving her best hopes would make in her life.

The practitioner then asks a follow-up question below (lines 2–3) to the best hopes question, “what difference will it make?” if Charlotte is mentally better. This helps her to articulate the positive difference this will make in her life. Charlotte responds, “a lot of difference”, and the practitioner asks for a description. Charlotte states she would be happy to go to school, socialise with people in school, and participate in lessons (lines 6–7) (Figure 4).

- Miracle Question

**Figure 5:**
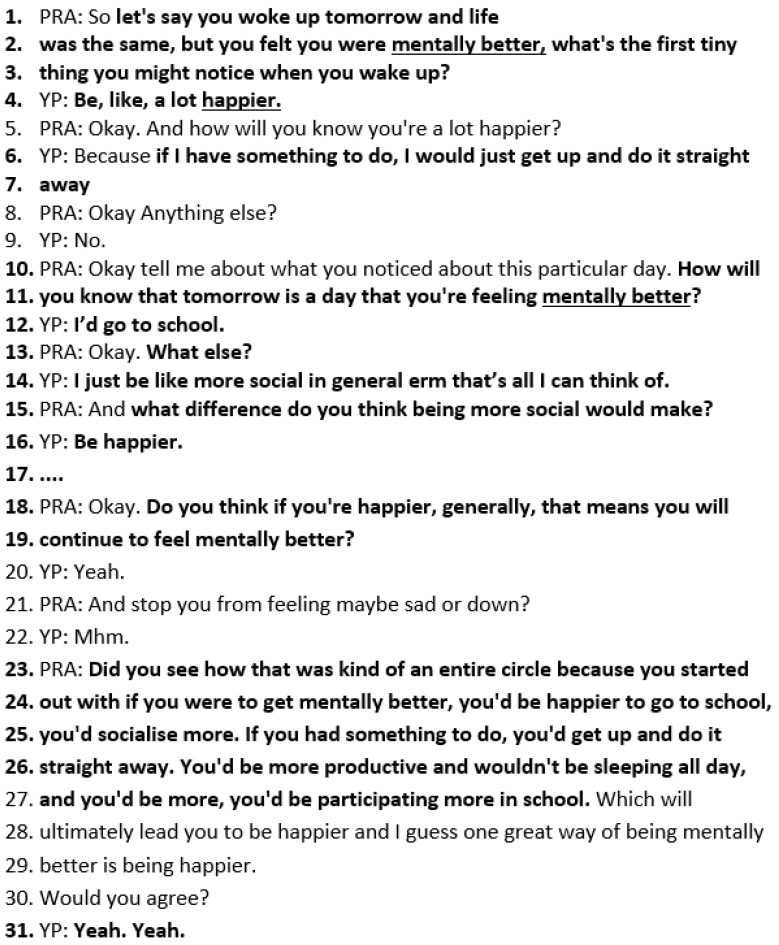
Transcript excerpt from Charlotte’s SFBT session: the miracle question.

After establishing the person’s best hopes, the next “miracle question” (lines 1–3 below) encourages Charlotte to *visualise a good day* when she is feeling mentally better. This prompts Charlotte to visualise a day when she is “mentally better” and provides descriptive details about what will be happening on this kind of day. Charlotte states she would be “a lot happier” (line 4). By asking “how will you know you’re a lot happier”, the practitioner elicits a behavioural description: “if I have something to do, I would just get up and do it straight away” (lines 6–7). A further question, “How will you know that tomorrow is a day that you’re feeling mentally better?” elicits further detail from Charlotte, “I’d go to school”. Although this is a very similar question to the previous miracle question in lines 1–3, it elicits further behavioural description about how this will show in her life. The practitioner then asks a common SF question, “what else” (line 13), to encourage further description. The YP adds she would be “more social in general” (line 14) (Figure 5).

The SF questioning prompts Charlotte to describe getting up straight away if she has something to do, going to school, being more social, and participating more in lessons, all of which would make her feel happier. Asking questions that elicit detailed descriptions translates what “mentally better” would look like in Charlotte’s day.

### 3.4. Case Study 4: Follow-Up Solution Focused Session with Wai

**Figure 6:**
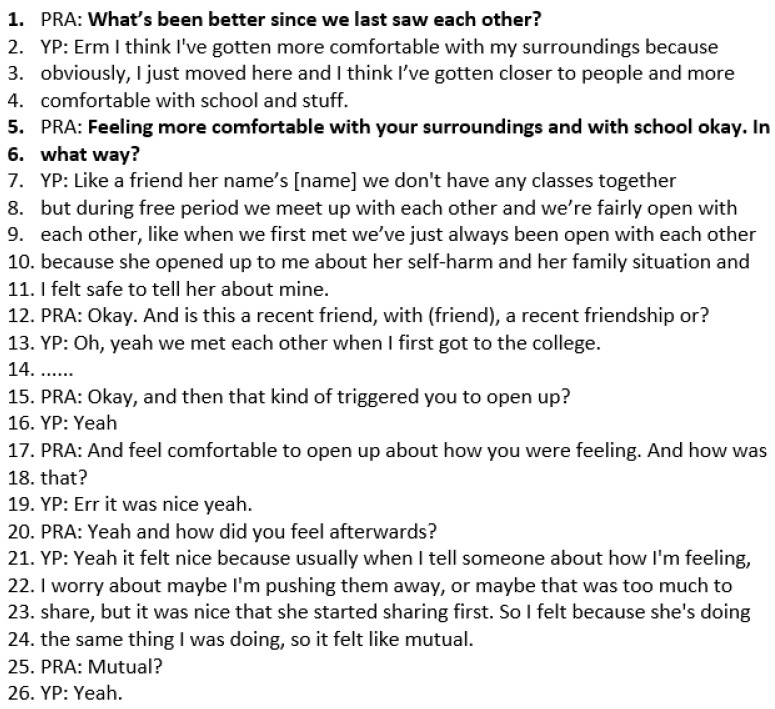
Transcript excerpt from Wai’s follow-up SFBT session: describing what has been better since the last session.

Follow-up SF sessions focus on what has been better and amplifying the person’s resources, coping skills, and strengths. This follow-up session is with a YP called Wai, who is aged 16. Reflecting on the impact of the solution-focused sessions, Wai said she has “been a lot more calm in stressful situations…” The opening question in a follow-up SF session is “What has been better?”. This directs Wai to *notice small signs of progress*, as described in the logic model. In the transcript below, Wai responds that she has become more comfortable with her surroundings, her school, and has got closer to people. The practitioner invites Wai to elaborate further in lines 5–6, “In what way?”. Wai describes meeting with a friend between classes and opening up to her about her self-harm and how she was feeling. Wai describes how it felt nice to share how she was feeling with her friend, as it was mutual because she usually worries about pushing people away or sharing too much (lines 21–24) (Figure 6).

**Figure 7:**
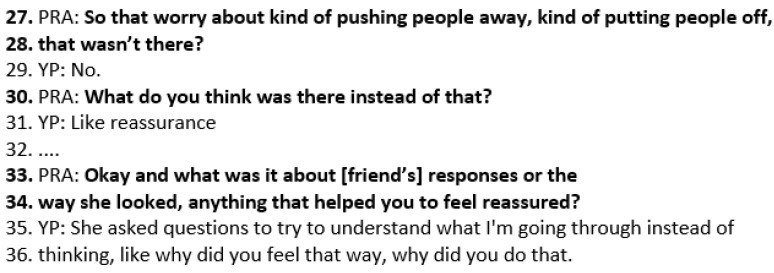
Transcript excerpt from follow-up SFBT session with Wai: exceptions to the problem.

When the practitioner asks Wai what was there instead of worry (line 30), the YP responds that there was reassurance. This draws out *exceptions to the problem*, namely how Wai is able to share how she feels and feel reassured rather than worried. The practitioner asks what it was about how Wai’s friend responded that helped her feel reassured (33–34). Wai describes how her friend tried to understand what she was going through rather than asking her why she felt that way or why she did what she did (Figure 7).

**Figure 8:**
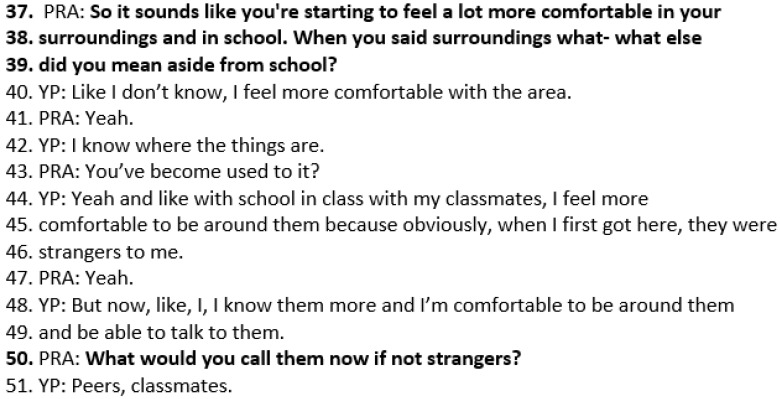
Transcript excerpt from Wai’s follow-up SFBT session: amplifying signs of progress.

The practitioner then returns to Wai’s description of being better at the beginning of the session, “feeling more comfortable with my surroundings”, and asks her to elaborate further. This activity is *amplifying signs of progress*. Wai describes feeling more comfortable with the area (as she had recently moved there), along with feeling more comfortable with her classmates and being around them and being able to talk to them. When Wai describes how other students were “strangers” at first (lines 46–47), the practitioner asks what she could call them now, rather than strangers. Wai responds, “peers, classmates”. These questions elicit Wai’s personalised descriptions of how “more comfortable” is happening in her life (Figure 8).

**Figure 9:**
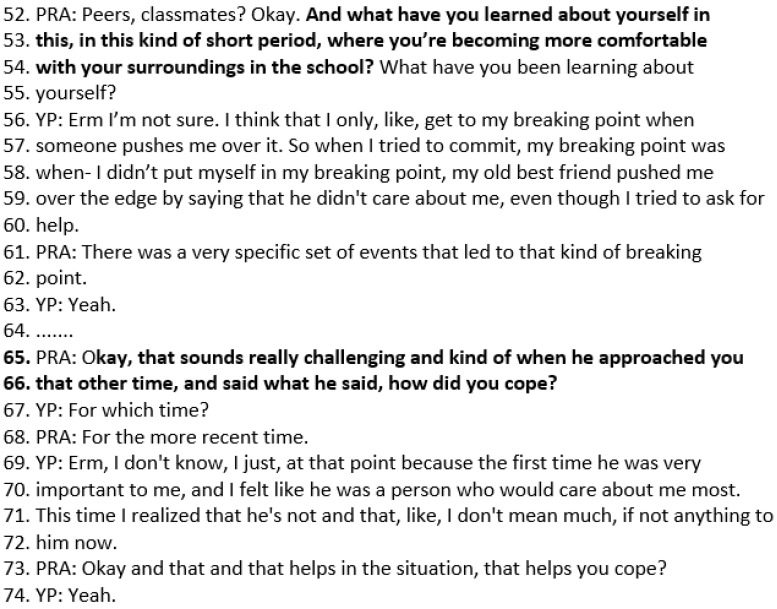
Transcript excerpt from Wai’s follow-up SFBT session: identifying coping strategies.

In the following excerpt, the practitioner asks Wai what she has learned about herself. Wai describes a specific set of events that led to her breaking point (lines 56–60) when her old best friend said he did not care about her. The practitioner then asks about *coping strategies:* “that sounds really challenging. Kind of when he approached you, and said what he said, how did you cope?” (lines 65–66). Wai describes that previously she felt that he was a person who cared about her and that she “realised that he’s not and that, like, I don’t mean much, if not anything to him now”. She describes this realisation as helping her not to be pushed to her breaking point (Figure 9).

- Scaling Questions

**Figure 10:**
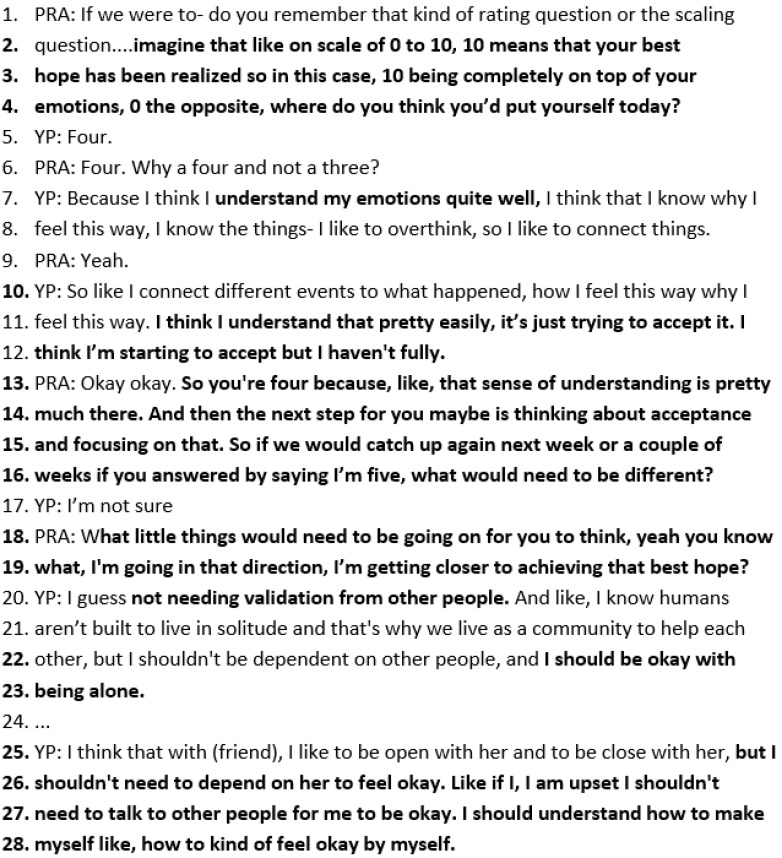
Transcript excerpt from Wai’s follow-up SFBT session: scaling question.

*To identify what is already working* is commonly used in SFBT. The practitioner asks in lines 2–4 below, “imagine that like on scale of 0 to 10, 10 means that your best hope has been realized so in this case, 10 completely, 0 the opposite where do you think you’d put yourself?”. Wai responds, “four”. The focus is on why Wai is where they are on the scale *and not lower*. Wai states that she understands her emotions quite well: She knows why she feels the way she does, and she overthinks. She is starting to accept it but has not fully accepted it fully yet. The practitioner reiterates that she is a “four” because of that “sense of understanding”, then explores *small signs of progress* by asking Wai what would be different if she were one point up—a five on the scale (lines 13–16). Wai describes not feeling like she needs validation or to talk to other people if she is feeling upset: Rather, she would be feeling okay by herself (lines 20–28) (Figure 10).

### 3.5. Case Study 5: Solution Focused Session with Carer Michelle

Sessions are offered to carers if they would like to avail of them. Sessions can be for one or more carers. In interviews, practitioners noted that these sessions gave a space for carers to talk, reflect on supporting themselves and their mental health, helped bridge understanding between them and their son/daughter, and sometimes were a space to reflect on their own experiences of growing up, their relationships with their parents/carers, along with diverse cultural approaches to parenting. Practitioners found that identifying where the YP and their carer have some shared aspect in their best hopes can provide a basis for improved relationships, which are often strained by the crisis. Given that relationship problems between family members are commonly reported problems in YP who self-harm [29], this may contribute to a reduction in the risk of repeated self-harm. A practitioner reflected on the impact of improved understanding among YP and carers and offering targeted support to carers: “every single dynamic of YP and parent I’ve worked with have had a transformed dynamic by the end of the intervention… the YP may be the one who is self-harming, but the parent is often also in crisis”.

**Figure 11:**
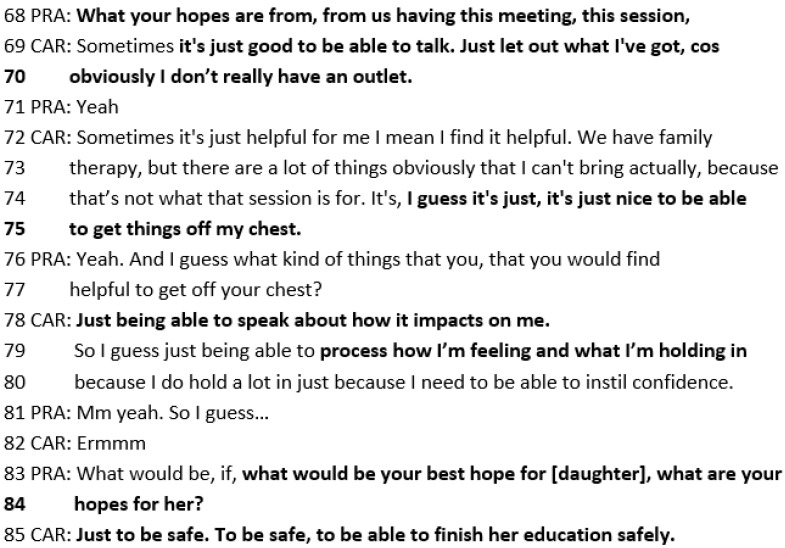
Transcript excerpt of parent/carer SFBT session with Michelle: Michelle’s best hopes.

In the following SFBT session with Michelle, she describes her best hopes of “having an outlet”, “being able to talk”, “get things off my chest”, “being able to speak about how it impacts on me”, and “process how I’m feeling and what I’m holding”. The practitioner also explores her best hopes for her daughter Wai (83–84), which are for her “to be safe” and “finish her education safely” (line 85) (Figure 11).

**Figure 12:**
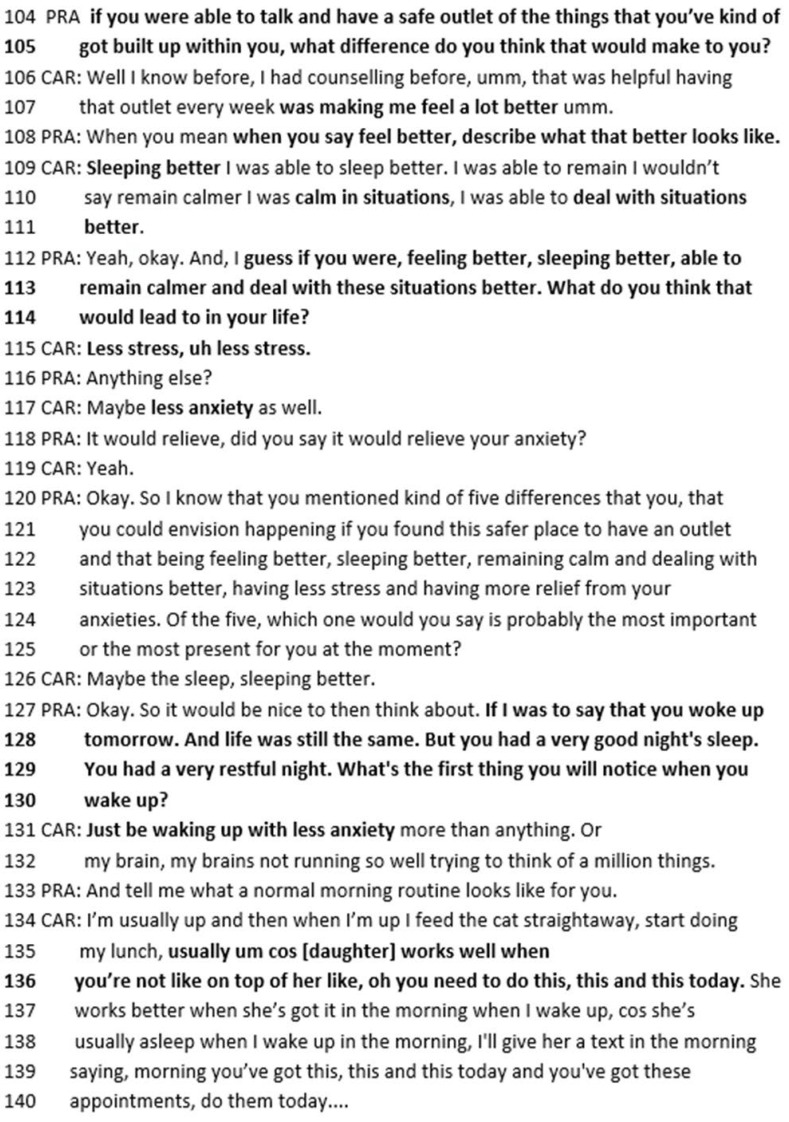
Transcript excerpt from Michelle’s follow-up SFBT session: exploring the difference achieving her best hopes would make.

The practitioner then asks, “what difference do you think that would make to you if you were able to talk and have a safe outlet?” (lines 104–105). In SFBT, it is very important to use the person’s exact words. This question helps Michelle to describe how the desired changes would impact tangibly on her life: This would lead to her feeling better. The practitioner asks Michelle to describe “what better looks like”. Michelle responds that it would be “sleeping better” and “being calm in situations”. Again, the practitioner uses Michelle’s words (“if you were feeling better, sleeping better, able to remain calmer and deal with these situations better, what do you think that would lead to in your life?”). Michelle describes “less stress” (115) and “less anxiety” (117). Michelle then describes her morning after she has slept better, including that her daughter “works well when you’re not on top of her” (135–136). If Michelle has slept better, this would also have an impact on her daughter in the morning before they start their (Figure 12).

**Figure 13:**
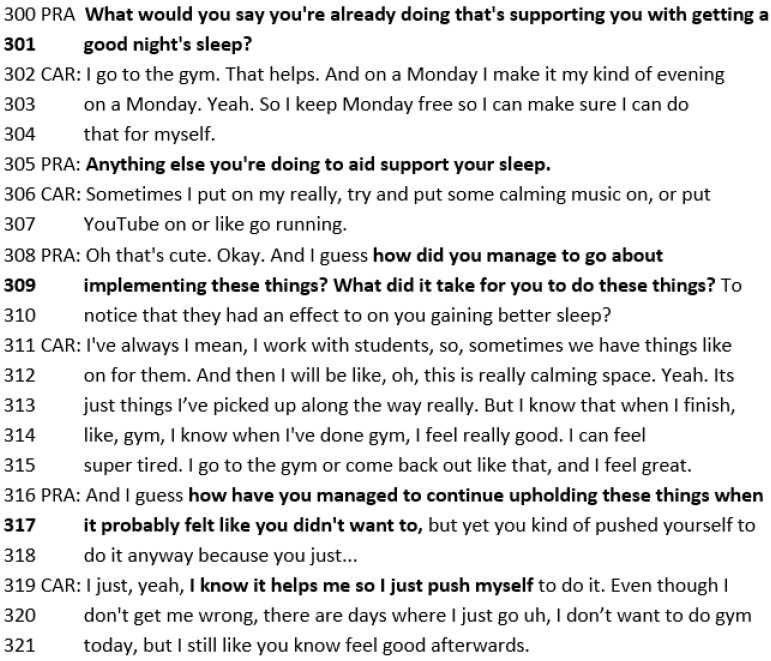
Transcript excerpt from Michelle’s follow-up SFBT session: encouraging Michelle to notice what is already working.

The practitioner then asks Michelle what she is already doing to get a good night’s sleep (lines 300–301 below). This directs Michelle to *notice what is already working.* Michelle reports going to the gym and having an evening for herself. When the practitioner asks, “Anything else”, this elicits further information about positive things Michelle is already doing: Michelle puts on calming music/YouTube and goes running. The practitioner then asks *strategy* questions: “how did you go about managing to implement these things? What did it take for you to do these things?” (lines 309–310). These questions elicit Michelle’s resources (“I know it helps me so I just push myself”—line 320) and what she is already doing to achieve her best hope of “sleeping better” so that she is “feeling better” (Figure 13).

These brief SFBT excerpts illustrate some of the techniques and questions that are used to focus on what the YP or carer wants to be better (their best hopes) and what will be happening if their best hopes are achieved (their preferred future). The conversations show how practitioners attend closely to the YP/carer’s exact words. This supports shared understanding and collaborating closely with the YP and carer in.

## 4. Discussion

The intervention materials and session transcripts illustrate how the SASH intervention can support YP to (1) better understand *their personal* self-harm and identify ways of breaking the cycle of self-harm, (2) develop a highly *personalised and granular safety plan* with a range of coping strategies, and (3) identify their *personal hopes* for what they want to be better in their lives and how they are already moving in this direction. All three components prioritise the YP’s or carer’s language and use their words to formulate the cycle of self-harm, the safety plan, and hopes for/progress towards their preferred future. The collaborative approach fosters the therapeutic relationship and personalised care.

Across the sessions, some core themes emerged. The role of friendships for young people, particularly at school, was important in both negative and positive ways. Experiencing difficulties with friends at school led to feelings of sadness and stress, which could become overwhelming, leading to thoughts of self-harm (“I just need to hurt myself”) and triggering self-harm behaviour. YP described noticing mood changes and signs that they were becoming stressed, which improved their self-awareness and understanding of the link between their feelings and self-harm behaviour. They reflected on what kept them feeling calm and overcoming a fear of being a burden on others by sharing how they were feeling, which helped them not to self-harm. They also described difficult feelings stemming from a need to please everyone or needing validation from others: Overcoming these feelings led to less social anxiety and more confidence. This led to finding it easier to go to school and being more social with friends/student peers, which in turn improved their mood

In the TA case study of Adah, drawing the cycle of self-harm helped the YP to unpack the “core pain” behind their self-harm (sadness) and how this led to self-harm (“I thought it was only option”). Breaking down the chain of events and asking the YP to consider the positive short-term consequences (relief as “nothing going on in my head”) and then the negative longer-term consequences (a panic attack and having to attend the ED) helps the YP to understand the link between their emotions and behaviour. Developing a detailed personalised trajectory enables the YP to explore where *they* feel it would be helpful to think about breaking the cycle. This contrasts with more generic advice about stopping self-harm and reflects the logic model mechanisms of change, namely building the YP’s self-awareness of their self-harm and instilling confidence in how to break the cycle. Synthesis of accounts of people who reduced or stopped self-harming reveals the importance of breaking the link between an individual’s psychological or social states and self-harm behaviour [30]. YP report being given advice that they do not feel is relevant for them [31]. Previous research has found the Therapeutic Assessment to be effective in improving engagement in subsequent follow-up care [32]. In this case, the YP attended four further sessions after the TA, indicating good engagement in the SASH intervention.

The SP case illustrates an initial focus on what the YP is like when they are well. This addition to the SP was suggested by our Young Person Advisory Group (YPAG) who we collaborated with during the SASH trial. This is consistent with a more positively framed approach, similar to the Reasons for Living approach, which focuses on positive reasons for staying alive in YP admitted to the hospital [33]. Enhanced Safety Planning is a very granular approach. YP identify *their* own personal warning signs (thoughts, emotions, and body sensations), where they typically are when these signs appear, and a range of coping strategies they can draw on in these moments. It incorporates principles of behaviour change by explicitly asking about barriers to implementing helpful behaviours and reaching out to others when things are starting to deteriorate and how to overcome these barriers.

While SP are recommended in NICE guidelines following an episode of self-harm [1], they typically provide basic information about means restriction and crisis phone numbers. Although there is little research on YP’s experiences of safety planning, adults have described safety plans as too generic and not sufficiently personalised to them [34]. Patients often report doubts about being able to use their safety plan [35]. This reflects the importance of troubleshooting barriers to using the coping strategies and thinking these through before the person is going into or in a crisis: Adah described saying positive affirmations to herself in these moments. These strategies provide new ways of thinking, which support the YP’s motivation and hope that they can break the self-harm cycle.

SFBT sessions focus on the YP’s or carer’s best hopes for the future (rather than problems) and current strengths. They generate positive emotions by visualising and describing a good day, identifying what is already working, noticing exceptions to the problem, and orienting the YP/carer to notice small signs of progress. Similar to TA and SP, SFBT elicits personalised, detailed descriptions about the YP/carer’s situation. In Case study 3, Charlotte describes wanting to get better mentally and to communicate better. Her visualisation and detailed description of a good day starts with her getting straight out of bed in the morning and being happier to go to school and socialise more with people. This would make her happier, which in turn would make her mentally better. The transcripts of the SFBT sessions show how questions elicit detailed descriptions of what will be happening in the person’s daily life when things are better [36].

SFBT has been used with people with suicidal thoughts or in a mental health crisis [37]. By focusing on the person’s strengths and resources, it helps to instil hope [37]. SF approaches have also been used in the assessment and [37,38] management of suicide risk, albeit more commonly in adults [39]. A solution-focused approach is a paradigm shift from the usual way of working in crisis mental health care in the NHS. However, it can be embedded within NHS systems of care and in the context of self-harm and suicide risk management [40].

Supporting carers is increasingly recognised as an important part of care for YP presenting to the ED with self-harm in a mental health crisis. Recent clinical and practice reviews recommend greater attention paid to family involvement [7,18]. Meanwhile, Mughal et al. (2022), in their systematic review of the needs of individuals supporting YP, found that they also need support, especially as they negotiate new identities when handling self-harm in YP [41].

### Strengths and Limitations

This paper presents five case studies based on materials and recordings of the SASH intervention sessions, which were collected as part of a process evaluation in an ongoing randomised controlled trial. The intervention was implemented in routine clinical settings in the NHS in the UK. Clinical and other outcomes (e.g., depression, anxiety, wellbeing, and school attendance) are assessed after 6 months, and the primary outcome is self-harm. The trial will also consider service use, costs to carer, and carer health-related quality of life to evaluate the costs and cost-effectiveness of the intervention.

As described by McLeod (2010) [27], this is useful for demonstrating how psychological interventions are delivered in practice and provides practitioners with real examples of working with YP presenting with self-harm. We used case materials from medical records and intervention recordings to illustrate components of the SASH intervention delivered by child and adolescent mental health practitioners. While the RCT results have not yet been published, this study offers insights into real-world treatment, providing fine-grained and practice-based evidence of how the SASH intervention was delivered to YP in crisis. This offers valuable information for clinicians on how the SASH techniques were implemented in real clinician-patient interactions. Multiple sources of data, including intervention session transcripts, bespoke intervention materials, and qualitative interviews, are used to demonstrate the delivery of the intervention in practice. The logic model provides a structured conceptual framework for interpreting potential mechanisms of change of the intervention. While we only presented five case studies, there was cultural diversity in the cases described.

Limitations relate to the availability of intervention session recordings and sampling of the cases. Intervention sessions were sampled according to availability, audio quality, and fidelity to the intervention according to the manual. Only *n* = 16 intervention sessions were recorded among 78 participants. Consent from the young person, carer, and practitioner was required, and in many cases, consent to recording was not given by one or all parties. Future studies could further encourage the recording of intervention sessions in research studies.

Only five case studies were presented, which may not capture the variation in implementation across different groups of YP and may not be representative of the broader pool of intervention sessions. Once the trial results are available, it will be important to consider the clinical and service use outcomes of the YP in the SASH intervention compared to those receiving Treatment As Usual. Given the link between ED self-harm presentations and the risk of future suicide, re-presentations to the ED will be of particular interest. In addition, data on YP engagement in the intervention, YP who dropped out, and the number of sessions attended will provide insights into the acceptability of the intervention. The SASH trial recruited YP and carers from nine EDs in London. All of the case studies were based on YP who were female (and cis gender). This reflects the overall SASH sample, which was predominantly female. None of the current case studies included YP who were neurodivergent or gender diverse. This may limit the generalisability of the findings to a more diverse group of YP and carers.

## 5. Conclusions

These case studies demonstrate how YP improved their self-awareness and understanding of the link between feelings and self-harm behaviour and identified ways of breaking the self-harm cycle, along with detailed strategies for managing difficult feelings and situations at school and with friends. The carer case study demonstrates the potential to support carer mental health so they can, in turn, better support their YP’s mental health. Supporting YP and carers in this way has the potential to reduce the risk of future self-harm.

## Figures and Tables

**Table 1:** Participant characteristics.

Pseudonym	YP/Carer	ED Presentation	Age	Gender	Neurodiversity	Ethnicity	No. Sessions Attended
Adah	YP	Suicide attempt	13	F	N	Mixed ethnic background	5
Eliana	YP	Suicide attempt	15	F	N	Black British	5
Charlotte	YP	Suicidal ideation (and recent self-harm)	14	F	N	White British	5
Wai	YP	Suicide attempt	16	F	N	Other—Hong Kong and Indonesia	4
Michelle	Carer	N/A	53	F	N	Black British	1

## Data Availability

The original contributions presented in this study are included in the article. Further inquiries can be directed to the corresponding author.

## Supplementary Materials

The following supporting information can be downloaded at: https://www.mdpi.com/article/10.3390/healthcare14020168/s1, Figure S1: Therapeutic Assessment: cycle of self-harm; Figure S2: enhanced safety plan template; Figure S3: solution-focused brief therapy template (first session); Figure S4: solution-focused brief therapy template (follow-up session).
