# Supplementary material for: The “Supporting Adolescents with Self Harm” (SASH) Intervention Supporting Young People (And Carers) Presenting to the Emergency Department with Self-Harm: Therapeutic Assessment, Safety Planning, and Solution-Focused Brief Therapy
Source: Healthcare (Basel). 2026 Jan 8;14(2):168. doi: 10.3390/healthcare14020168 (PMC12840726; doi:10.3390/healthcare14020168)
Supplement: Supplementary file 1 [file healthcare-14-00168-s001.zip › healthcare-4005450-supplementary.pdf]

Supplementary materials

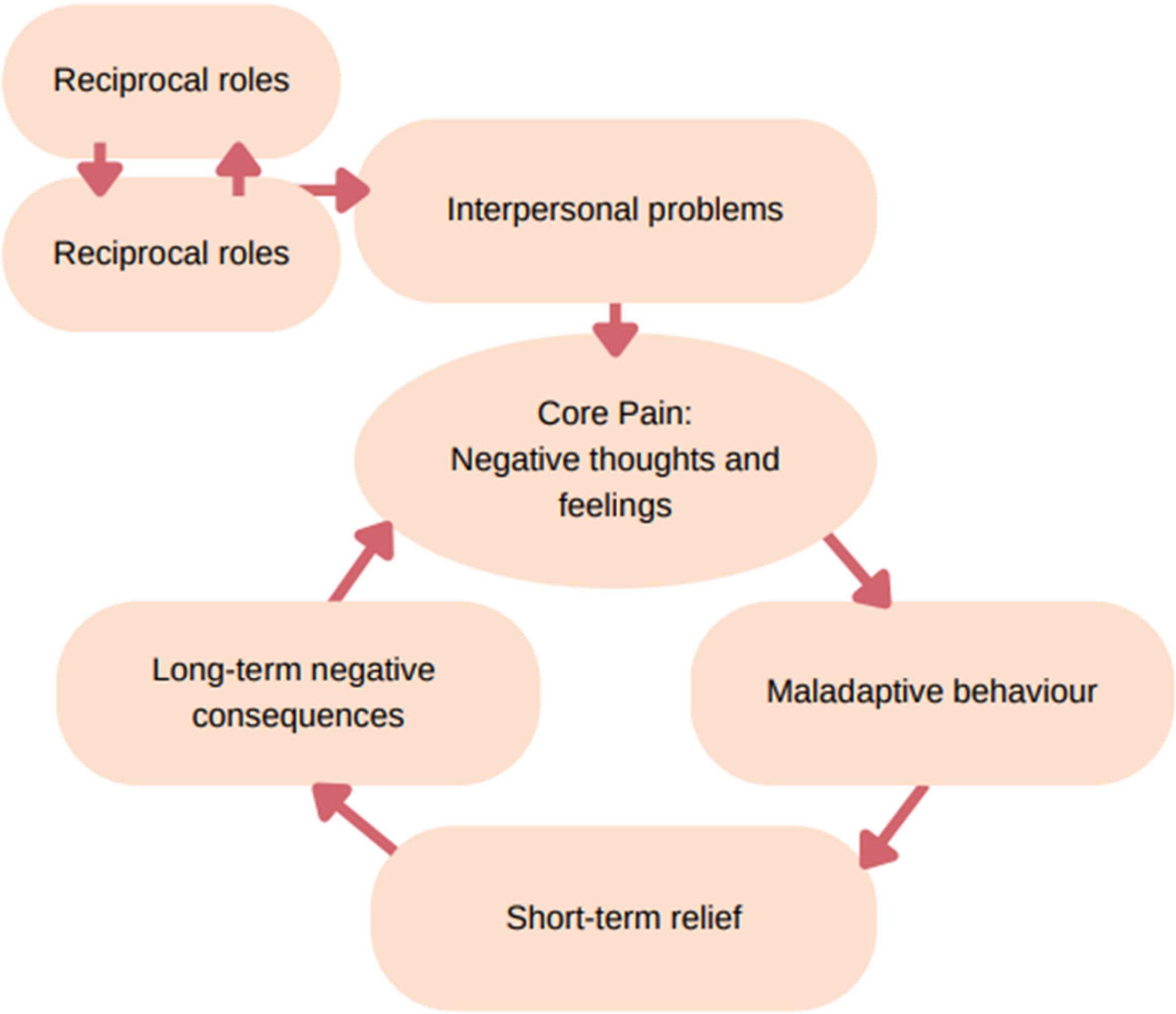

Figure S1. Therapeutic Assessment: Cycle of Self-Harm

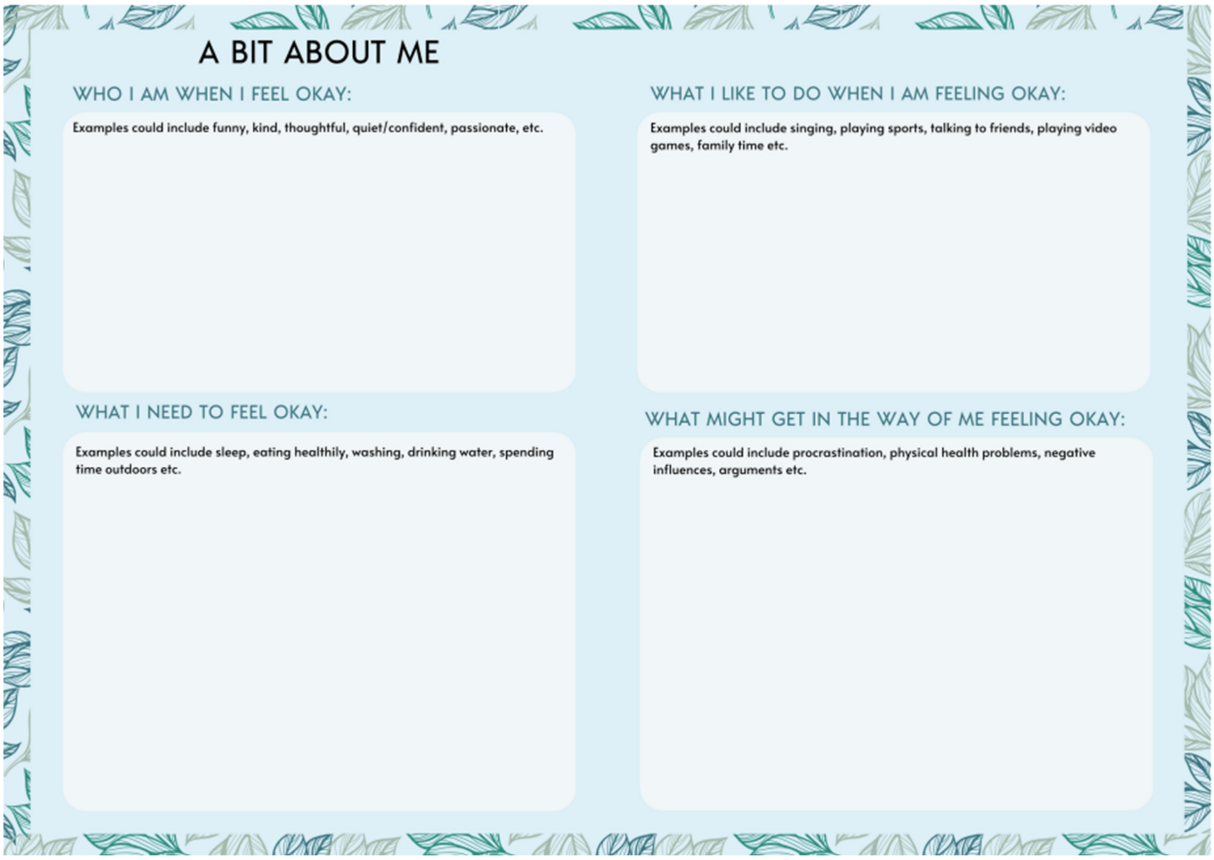

A BIT ABOUT ME

WHO I AM WHEN I FEEL OKAY:

Examples could include funny, kind, thoughtful, quiet/confident, passionate, etc.

WHAT I LIKE TO DO WHEN I AM FEELING OKAY:

Examples could include singing, playing sports, talking to friends, playing video games, family time etc.

WHAT I NEED TO FEEL OKAY:

Examples could include sleep, eating healthily, washing, drinking water, spending time outdoors etc.

WHAT MIGHT GET IN THE WAY OF ME FEELING OKAY:

Examples could include procrastination, physical health problems, negative influences, arguments etc.

(**a**)

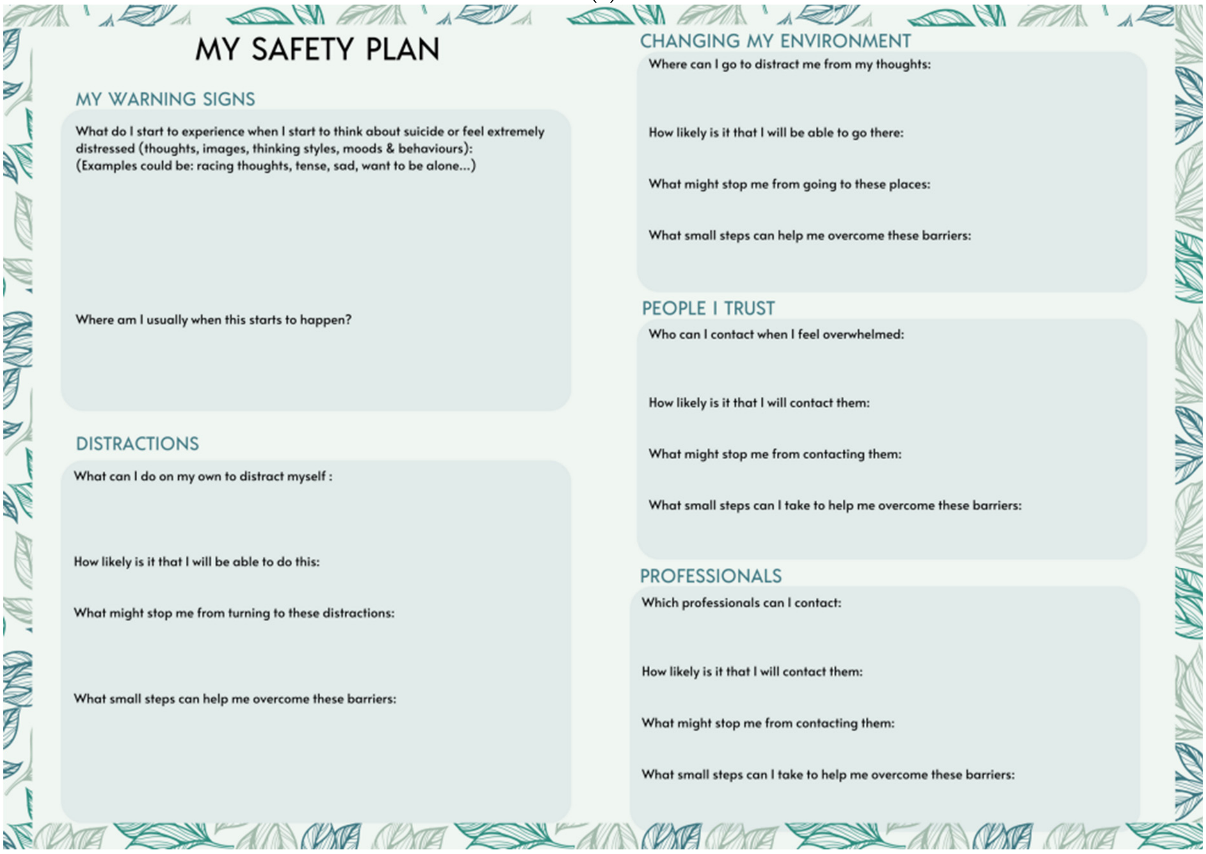

MY SAFETY PLAN

MY WARNING SIGNS

What do I start to experience when I start to think about suicide or feel extremely distressed (thoughts, images, thinking styles, moods & behaviours):
(Examples could be: racing thoughts, tense, sad, want to be alone...)

Where am I usually when this starts to happen?

DISTRACTIONS

What can I do on my own to distract myself :

How likely is it that I will be able to do this:

What might stop me from turning to these distractions:

What small steps can help me overcome these barriers:

CHANGING MY ENVIRONMENT

Where can I go to distract me from my thoughts:

How likely is it that I will be able to go there:

What might stop me from going to these places:

What small steps can help me overcome these barriers:

PEOPLE I TRUST

Who can I contact when I feel overwhelmed:

How likely is it that I will contact them:

What might stop me from contacting them:

What small steps can I take to help me overcome these barriers:

PROFESSIONALS

Which professionals can I contact:

How likely is it that I will contact them:

What might stop me from contacting them:

What small steps can I take to help me overcome these barriers:

(**b**)

Figure S2. Enhanced Safety Plan Template

Solution Focused handout

Date: Participant name: Practitioner initials:

Session number:

Duration of session in minutes:

Identifying "Better"

What I would like to be better/ different

1

2

3

What difference will this make?

If I woke up tomorrow and things were better/ different, what would I notice first?

What else?

Who might be the first person I see on this day?

What might they notice?

How will I know they noticed?

How would I respond?

What difference would that make to me?

In the last few days/ weeks what, even small things, am I already doing that's working? What strengths/ resources do I already have?

What has happened recently to give me hope that things could be the way I'd like them to be?

Given how tough things have been, how have I been coping?

What does that tell me about myself?

[continue on next page]

Figure S3. Solution-Focused Brief Therapy Template (First Session)

Date: Participant name: Practitioner initials:

Session number:

Duration of session in minutes:

Better scale

Opposite of better — Better

0 ——————— 10

If 10 is my ideal 'better', and 0 is the opposite, **where am I now on the scale?** *(please mark)*

What tells me it's at ___ and *not lower*?

What is it that has *helped* me to get from 0 to where I am now? What is stopping things from getting even worse?

How am I managing to hold things where they are even though they are so difficult?

How will I know things have *improved* for me? What are *small signs of progress* I could look out for?

Confidence scale

No confidence — Definite confidence

0 ——————— 10

If 0 is having no confidence at all that I can move up one point towards my 'Better' and 10 represents knowing, tough though it may be, that I *definitely* will make progress, **where is my confidence now on the scale**? *(please mark)*

What tells me it's at ___ and *not lower*?

What do *you* know about yourself that tells you there is a *chance* of progress for you?

What do *others* know about you that tells them there is a chance of progress for you?

Final reflection

What have I learned about myself since working together?

My next session is on...

Figure S4. Solution-Focused Brief Therapy Template (Follow-up Session)
